# LSTMVoter: chemical named entity recognition using a conglomerate of sequence labeling tools

**DOI:** 10.1186/s13321-018-0327-2

**Published:** 2019-01-10

**Authors:** Wahed Hemati, Alexander Mehler

**Affiliations:** Text Technology Lab, Goethe-University Frankfurt, Robert-Mayer-Straße 10, 60325 Frankfurt am Main, Germany

**Keywords:** BioCreative V.5, CEMP, CHEMDNER, BioNLP, Named entity recognition, Deep learning, LSTM, Attention mechanism

## Abstract

**Background:**

Chemical and biomedical *named entity recognition* (NER) is an essential preprocessing task in *natural language processing*. The identification and extraction of named entities from scientific articles is also attracting increasing interest in many scientific disciplines. Locating chemical named entities in the literature is an essential step in chemical text mining pipelines for identifying chemical mentions, their properties, and relations as discussed in the literature. In this work, we describe an approach to the BioCreative V.5 challenge regarding the recognition and classification of chemical named entities. For this purpose, we transform the task of NER into a sequence labeling problem. We present a series of sequence labeling systems that we used, adapted and optimized in our experiments for solving this task. To this end, we experiment with hyperparameter optimization. Finally, we present LSTMVoter, a two-stage application of *recurrent neural network*s that integrates the optimized sequence labelers from our study into a single ensemble classifier.

**Results:**

We introduce LSTMVoter, a bidirectional *long short-term memory* (LSTM) tagger that utilizes a conditional random field layer in conjunction with attention-based feature modeling. Our approach explores information about features that is modeled by means of an attention mechanism. LSTMVoter outperforms each extractor integrated by it in a series of experiments. On the BioCreative IV chemical compound and drug name recognition (CHEMDNER) corpus, LSTMVoter achieves an F1-score of 90.04%; on the BioCreative V.5 chemical entity mention in patents corpus, it achieves an F1-score of 89.01%.

**Availability and implementation:**

Data and code are available at https://github.com/texttechnologylab/LSTMVoter.

## Introduction

In order to advance the fields of biological, chemical and biomedical research, it is important to stay on the cutting edge of research. However, given the rapid development of the disciplines involved, this is difficult, as numerous new publications appear daily in biomedical journals. In order to avoid repetition and to contribute at least at the level of current research, researchers rely on published information to inform themselves about the latest research developments. There is therefore a growing interest in improved access to information on biological, chemical and biomedical data described in scientific articles, patents or health agency reports. In this context, improved access to chemical and drug name mentions in document repositories is of particular interest: it is these entity types that are most often searched for in the PubMed [1] database. To achieve this goal, a fundamental preprocessing step is to automatically identify biological and chemical mentions in the underlying documents. Based on this identification, downstream NLP tasks such as the recognition of interactions between drugs and proteins, of side effects of chemical compounds and their associations with toxicological endpoints or the investigation of information on metabolic reactions can be carried out.

For these reasons, NLP initiatives have been launched in recent years to address the challenges of identifying biological, chemical and biomedical entities. One of these initiatives is the BioCreative series, which focuses on biomedical text mining. BioCreative is a “Challenge Evaluation”, in which the participants are given defined text mining or information extraction tasks in the biomedical and chemical field. These tasks include *GeneMention detection (GM)* [2, 3], *Gene Normalization (GN)* [3–5], *Protein–Protein Interaction (PPI)* [6], *Chemical Compound and Drug Name Recognition (CHEMDNER)* [7, 8] and *Chemical Disease Relation Extraction* [9, 10] tasks.

The current *BioCreative V.5* task consists of two off-line tasks, namely *Chemical Entity Mention in Patents (CEMP)* and *Gene and Protein Related Object Recognition (GPRO)*. CEMP requires the detection of chemical named entity mentions. The task requires detecting the start and end indices corresponding to chemical entities. The GPRO task requires identifying mentions of gene and protein related objects in patent titles and abstracts [11]. In this work, we focus on the CEMP task. The CEMP task is an abstraction of the common named entity recognition (NER) tasks, which can be reduced to a sequence labeling problem, where the sentences are represented as sequences of tokens. The task is then to tag chemical entity mentions in these sequences. The settings of the CEMP task are similar to the chemical entity mention recognition (CEM) subtask of CHEMDNER challenge in BioCreative IV [7]. Therefore, we addressed both tasks and their underlying corpora in our experiments. Note that the current article describes an extension of previous work [12].

The article is organized as follows: First we describe our methodical apparatus and resources. This includes the data and corpora used in our experiments. Then, we introduce state-of-the-art tools for NER and explain how we adapted them to perform the CEMP task. Next, we present a novel tool for combining NER tools, that is, the so-called LSTMVoter. Finally, we present our results, conclude and discuss further work.

## Materials and methods

In this section, we first describe the datasets used in our experiments. Then, the two-stage application of LSTMVoter is introduced.

### Datasets

In our experiments, two corpora of the BioCreative Challenge were used: the CHEMDNER Corpus [13] and the CEMP Corpus [14].

The CHEMDNER corpus consists of 10,000 abstracts of chemistry-related journals published in 2013. Each abstract was human annotated for chemical mentions. The mentions were assigned to one of seven different subtypes (ABBREVIATION, FAMILY, FORMULA, IDENTIFIER, MULTIPLE, SYSTEMATIC, and TRIVIAL). The BioCreative organizer divided the corpus into training (3500 abstracts), development (3500 abstracts) and test (3000 abstracts) sets.

For CEMP task, the organizers of *BioCreative V.5* provided a corpus of 30,000 patent abstracts from patents published between 2005 and 2014. These abstracts are divided into training (21,000 abstracts) and test (9000 abstracts) sets. The corpus is manually annotated with chemical mentions. For the construction of the CEMP corpus the annotation guidelines of CHEMDNER were used. Therefore, CEMP contains the same seven chemical mention subtypes as CHEMDNER. Table 1 shows the number of instances for both corpora for each of these subtypes.

**Table 1:** Number of instances for each subtype of CEMP and CHEMDNER corpus

Annotation	CEMP	CHEMDNER
Abbreviation	1373	9059
Family	36,238	8313
Formula	6818	8585
Identifier	278	1311
Multiple	418	390
Systematic	28,580	13,472
Trivial	25,927	17,802
No class	0	72
Total count	99,632	59,004

Both corpora were enriched with additional linguistic features. For this, multiple preprocessing steps were applied on each set including sentence splitting, tokenization, lemmatization and fine-grained morphological tagging by means of Stanford CoreNLP [15] and TextImager [16]. In addition, tokens were split on non-alphanumeric characters, as this variant brought a performance increase. Since the chemical mention detection task can be reduced to a sequence labeling problem, the corpora were converted into a sequence structure. To this end, a sequence of documents with sequences of sentences each containing a sequence of tokens was constructed and transformed according to a TSV format. Each word and its associated features are in one line separated by tabs. Sentences are separated by an empty line. For the labeling of the mentions, the IOB tagging scheme [17] was used (I = *inside of an entity*, O = *outside of an entity*, B = *beginning of an entity*). IOB allows the annotation of entities that span multiple tokens, where the beginning and the end of the entity is marked. This enables models to learn transition probability. LSTMVoter needs four datasets for the training process. Two pairs of training and development sets are required. Each pair is needed in one of the two stages of LSTMVoter (see section “System description”). Therefore, we divided the training set of CEMP into two series of training, development and test sets (each half of the original training set was split according to the pattern 60%/20%/20%), where the first series is used for stage one, and the second for stage two. For the CHEMDNER corpus the available training and development sets were joined and split into training and development sets according to the schema 80%/20%—as before, we distinguish two such series. For evaluating our classifiers with respect to CHEMDNER, the test set provided by the organizers of the challenge was used. For the following experiments we used the corpora described as so far.

### System description

In this section we describe our system. Our approach implements a two-stage application of long short-term memory (LSTM) using a conglomerate of sequence labelers for the detection of chemical mentions.

In the first stage, we trained and optimized five tools for NER for tackling this task, namely *Stanford Named Entity Recognizer* [18], *MarMoT* [19], *CRF++* [20], *MITIE* [21] and *Glample* [22]. For each of them, we optimized the corresponding hyperparameter settings. Generally speaking, hyperparameter tuning is a challenging task in machine learning. The optimal set of hyperparameters depends on the model, the dataset and the domain [23]. Our experiments focused on optimizing the hyperparameters of each NER system independently, which led to a noticeable increase in F-score compared to the default settings. For each NER, we performed the Tree-structured Parzen Estimator (TPE) [24] with 200 iterations. The results of the best performing model for each of these NER is listed in Table 2.

The NER tools are more or less independent of each other in the sense that one can find a subset of test cases that are correctly processed by one of them, but not by another. Therefore, combining these NERs is a promising candidate for increasing performance. We started with computing combinations of these NERs by means of a simple majority vote [25], where the target label is selected, that is assigned by the majority of classifiers. Our experiments show that a simple majority vote brings no gain in performance compared to the best performing reference systems being examined in our study (see Table 2). Thus, we developed a two-stage model, the so-called LSTMVoter, which trains a recurrent neural network (RNN) with attention mechanism to learn the best combination of the underlying sequence labeling tools from stage one.

**Fig. 1:**
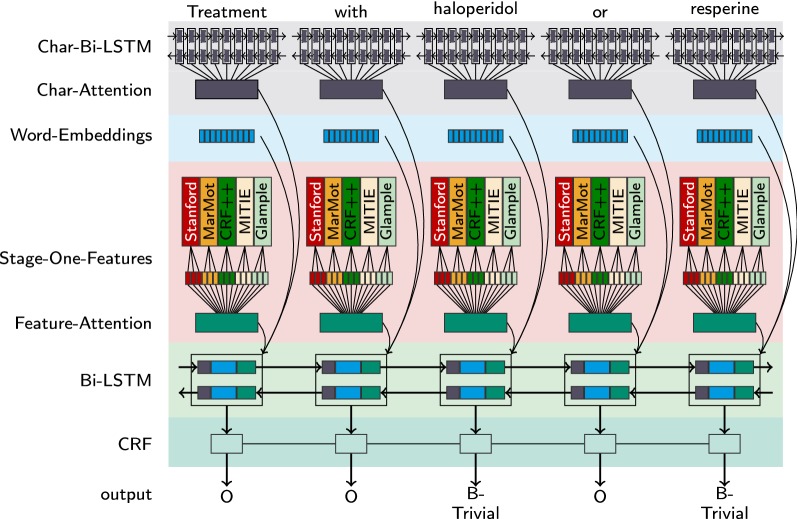
Architecture of LSTMVoter

In the second stage, we combine the sequence labelers of stage one with two bidirectional *long short-term memory* (LSTM) networks with attention mechanism and a conditional random field (CRF) network to form LSTMVoter. The architecture of LSTMVoter is illustrated in Fig. 1. The core of LSTMVoter is based on [22].

**Fig. 2:**
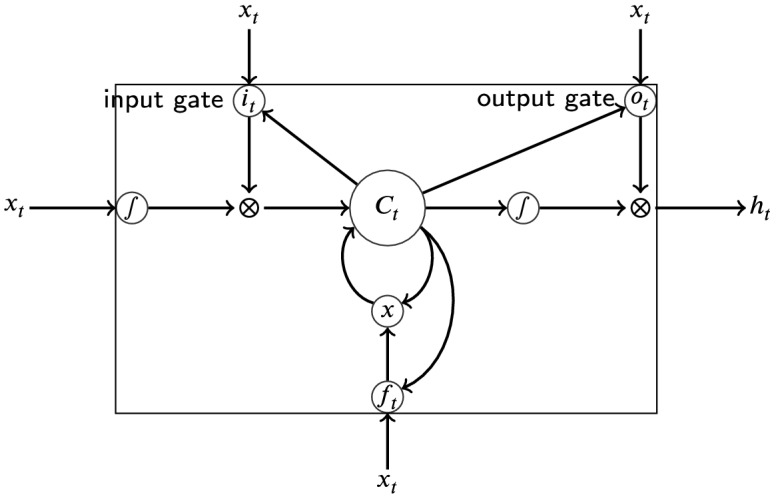
A long short-term memory cell

**Fig. 3:**
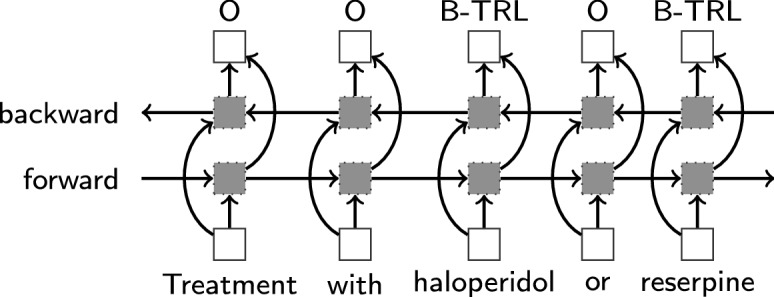
A bidirectional LSTM network

LSTM networks are a type of RNN [26]. RNN allow the computation of fixed-size vector representations for sequences of arbitrary length. An RNN is, so to speak, a function that reads an input sequence $x_1, \ldots , x_n$ of length *n* and produces an output vector $h_n$, which depends on the entire input sequence. Though, in theory, an RNN is capable of capturing long-distance dependencies in the input sequence, in practice, they may fail due to the problem of vanishing gradients [27, 28]. On the other hand, LSTMs include a memory cell, which can maintain information in memory for long periods of time [29, 30]. This enables finding and exploiting long range dependencies in the input sequences to cope with the problem of vanishing gradients. Figure 2 illustrates an LSTM memory cell, which is implemented as follows:

$$\begin{aligned} i_t&= \sigma (W_{xi}x_t + W_{hi}h_{t-1} + W_{ci}c_{t-1} + b_i)\\ f_t&= \sigma (W_{xf}x_t + W_{hf}h_{t-1} + W_{cf}c_{t-1} + b_f )\\ c_t&= f_tc_{t-1} + i_t \tanh (W_{xc}x_t + W_{hc}h_{t-1} + b_c)\\ o_t&= \sigma (W_{xo}x_t + W_{ho}h_{t-1} + W_{co}c_t + b_o)\\ h_t&= o_t \tanh (c_t) \end{aligned}$$

where $x_t$ is the input vector (e.g. word embedding) at time *t*. $h_t$ is the hidden state vector, also called output vector, that contains information at time *t* and all time steps before *t*. $\sigma$ is the logistic sigmoid function [31]. Input gate *i*, forget gate *f*, output gate *o* and cell vector *c* are of the same size as the hidden state vector *h*. $W_{hi}$, $W_{hf}$, $W_{hc}$ and $W_{ho}$ are the weight matrices for the hidden state $h_t$. $W_{xi}$, $W_{xf}$, $W_{xc}$ and $W_{xo}$ denote the weight matrices of different gates for input $x_t$.

For LSTMVoter, we apply an LSTM to sequence tagging. Additionally, as proposed by [32], we utilize bidirectional LSTM networks. Figure 3 illustrates a bidirectionalLong short-term memory (Bi-LSTM) network, where the input sequence (*Treatment with haloperidol or reserpine ...*) and the output sequence (*O, O, B-Trivial, O, B-Trivial, ...*) are fed as a training instance to a Bi-LSTM. In Bi-LSTMs, the input sequence is presented forward and backward to two separate hidden states to capture past and future information. To efficiently make use of past features (via forward states) and future features (via backward states) for a specific time frame, the two hidden states are concatenated to form the final output. In the final output of a Bi-LSTM, all information of the complete sequence is compressed into a fixed-length hidden state vector, which may result in information loss. To overcome this information loss, an attention mechanism is introduced, which partially fixes the problem.

The method of attention mechanism has recently gained popularity in image caption generation [33], visual question answering [34] and language modeling tasks [35–38]. The attention mechanism plugs a context vector on top of a layer, which enables to take all cells’ outputs as input to compute a probability distribution. This enables to capture global information rather then to infer based on one output vector.

For LSTMVoter, we utilized Bi-LSTM with attention mechanism to model character-level features (see Fig. 1, *Char-Bi-LSTM*). Character-level features in chemical named entities contain rich structure information, such as prefix, suffix and n-grams. Unlike previous methods [39–41], character-level features do not have to be defined manually, rather they can be learned during training. Unlike [22], who encodes the entire character sequence into a fixed-size vector for each word, we utilize the character-level attention mechanism introduced by [36]. This has the advantage, that by using the attention mechanism, the model is able to dynamically decide how much information and which part of a token to use.

In addition to the character-level features, we implemented word embeddings into our model to capture dependencies between words (see Fig. 1, *Word-Embeddings*). For this, we evaluated various methods, namely GloVe [42], Dependency-Based embeddings [43, 44] trained on the English Wikipedia, and word2vec [45] trained on the English Wikipedia and a biomedical scientific literature corpus containing PubMed abstracts and full texts. In our experiments, the word2vec model trained on biomedical scientific literature gave the best results.

To utilize the results of the NERs from stage one, we encode the respective results of the NERs into one-hot vectors concatenated to a feature vector (see Fig. 1, *Stage-One-Features*). An attention mechanism is placed on the feature vector. By creating a probability distribution through the attention mechanism, LSTMVoter learns how to weight each result of the NERs from stage one. With the attention vector it is even possible to determine for each element of a sequence how important the individual partial results from stage one were. This has the advantage that the model is no longer a black box, but can be interpreted as to how important the individual results from stage one were.

All previous elements of LSTMVoter encode word-based information. Another Bi-LSTM is used to learn relationships between these word-based information (see Fig. 1, *Bi-LSTM*).

To deal with the independent label output problem, we utilize the output vector as elements. For this we combine the Bi-LSTM layer with a linear-chain CRF (see Fig. 1, *CRF*). Linear-chain CRFs define the conditional probability of a state sequence to be:

$$\begin{aligned} P(y|x) = \frac{1}{Z_x}exp\left( \sum \limits _{j=1}^n \sum \limits _{m=1}^l \lambda _m f_m(y_{j-1},y_j,x,j)\right) \end{aligned}$$

where $Z_x$ is the normalization factor that makes the probability of all state sequences sum to one; $f_m(y_{j-1},y_j,x,j)$ is a feature function, and $\lambda _m$ is a learned weight associated with feature $f_m$. Feature functions measure the aspect of a state transition, $y_{j-1},y_j \rightarrow y_t$, and the entire observation sequence, *x*, centered at the current time step, *j*. Large positive values for $\lambda _m$ indicate a preference for such an event, whereas large negative values make the event unlikely.

Finally, to optimize the hyperparameters, the Tree Structure Parzen estimator was used.

## Results

This section presents the results of our experiments for the chemical named entity recognition on CEMP and CHEMDNER corpus. For evaluation the BioCreative Team has specified standard evaluation statistics, namely precision (P), recall (R) and F1-score (F) [14]. For each sequence labeling tool, the hyperparameters were optimized using Tree Structure Parzen Estimators, which led to a noticeable increase of performance. For example, in the optimization process of CRF++, the difference between the worst to the best performer is 65%. The results show the need for machine learning algorithms to perform hyperparameter optimization.

**Table 2:** Comparison of annotators trained and tested on CEMP and CHEMDNER corpora measured by precision (P), recall (R), f1-score (F1)

System	CEMP			CHEMDNER		
	P	R	F	P	R	F
Stanford NER	0.85	0.80	0.82	0.82	0.83	0.82
MarMoT	0.87	0.86	0.86	0.85	0.85	0.85
CRF++	0.77	0.73	0.73	0.74	0.71	0.73
MITIE	0.65	0.65	0.65	0.62	0.61	0.62
Glample	0.76	0.79	0.77	0.82	0.84	0.83
Majority vote	0.78	0.79	0.78	0.70	0.76	0.73
LSTMVoter	0.90	0.88	**0.89**	0.91	0.90	**0.90**

Bold was intended to compare LSTMVoter to the best reference tool. Bold now shows the system with the highest F-Score, which is LSTMVoter

Table 2 shows the comparison of annotators trained on CEMP and CHEMDNER corpus. The results listed are those obtained after the hyperparameter optimization described in the methods section, which were trained, optimized and tested on the corpora described in this section. Each sequence labeling system classifies a different subset correctly. The combination of sequence labelling systems in a majority vote did not improve performance and is even below the best sequence labelling systems. In contrast, LSTMVoter increases the performance and performs best in our experiments.

## Conclusions

In this work, we compared a set of sequence labeling systems. We trained and optimized every sequence labeling system to detect chemical entity mention by means the TPE. We showed that optimizing hyperparameter can be crucial. One sequence labeling system in our experiments gained an improvement of more than 65 %. We showed that a naive majority vote does not bring any improvement. For this reason, we introduced and evaluated LSTMVoter, a two-stage tool for combining underlying sequence modeling tools (as given by the NER of our comparative study). LSTMVoter achieved an improvement of up to 5 % compared to the best reference systems examined in our study. This two-level classifier appears to be capable of being further developed and improved by feeding it with the output of additional sequence labelling systems. In any event, our results and those of the other participants of BioCreative V.5 Task show that the task of NER of chemical entities has not been sufficiently solved yet. For a better recognition, a larger corpus should be generated so that today’s popular deep learning algorithms can work on this data. A kind of human-in-the-loop architecture for automatic annotation and intellectual rework would also be helpful at this point in order to successively increase and improve the amount of data.

## Abbreviations

Bi-LSTM: bidirectional long short-term memory
CEM: chemical entity mention recognition
CEMP: chemical entity mention in patents
CHEMDNER: chemical compound and drug name recognition
CRF: conditional random field
F: F1-score
GM: gene mention detection
GN: gene normalization
GPRO: gene and protein related object recognition
LSTM: long short-term memory
NER: named entity recognition
P: precision
PPI: protein–protein interaction
R: recall
RNN: recurrent neural network
TPE: tree-structured Parzen estimator
